# Gene and Protein Expression in Subjects With a Nystagmus-Associated AHR Mutation

**DOI:** 10.3389/fgene.2020.582796

**Published:** 2020-09-24

**Authors:** Natalia Borovok, Celeste Weiss, Rajech Sharkia, Michal Reichenstein, Bernd Wissinger, Abdussalam Azem, Muhammad Mahajnah

**Affiliations:** ^1^Faculty of Life Sciences, School of Neurobiology, Biochemistry and Biophysics, Tel Aviv University, Tel Aviv, Israel; ^2^Triangle Research and Development Center, Kafr Qara, Israel; ^3^Beit Berl College, Beit Berl, Israel; ^4^Institute for Ophthalmic Research Centre for Ophthalmology, Eberhard Karls University of Tübingen, Tübingen, Germany; ^5^Hillel Yaffe Medical Center, Hadera, Israel; ^6^The Ruth and Bruce Rappaport Faculty of Medicine, Technion – Israel Institute of Technology, Haifa, Israel

**Keywords:** aryl hydrocarbon receptor, human mutation, infantile nystagmus, gene expression, protein expression, CYP1A1, CYP1B1, TiPARP

## Abstract

Recently, a consanguineous family was identified in Israel with three children affected by Infantile Nystagmus and Foveal Hypoplasia, following an autosomal recessive mode of inheritance. A homozygous stop mutation c.1861C > T; p.Q621^∗^ in the aryl hydrocarbon receptor (*AHR*) gene (*AHR*; MIM 600253) was identified that co-segregated with the disease in the larger family. AHR is the first gene to be identified causing an autosomal recessive Infantile Nystagmus-related disease in humans. The goal of this study is to delineate the molecular basis of this newly discovered human genetic disorder associated with a rare *AHR* gene mutation. The gene and protein expression levels of *AHR* and selected *AHR* targets from leukocyte cultures of healthy subjects and the patients were analyzed. We observed significant variation between mRNA and protein expression of CYP1A1, CYP1B1, and TiPARP under rest and AHR-induced conditions. The CYP1A1 enzymatic activity in induced leukocytes also differs significantly between the patients and healthy volunteers. Intriguingly, the heterozygous subjects demonstrate CYP1A1 and TiPARP gene and protein expression similar to homozygous patients. In contrast, CYP1B1 inducibility and expression vary between hetero- and homozygous subjects. Similarity and differences in gene and protein expression between heterozygotes and homozygous patients can give us a hint as to which metabolic pathway/s might be involved in the Nystagmus etiology. Thus, we have a unique human model for AHR deficiency that will allow us the opportunity to study the biochemical basis of this rare human mutation, as well as the involvement of AHR in other physiological processes.

## Introduction

We recently described a consanguineous Israeli Arab family with three children affected by Idiopathic Infantile Nystagmus (IIN) and foveal hypoplasia, due to a homozygous stop mutation c.1861C > T; p.Q621^∗^ in the gene encoding the aryl hydrocarbon receptor (*AHR*; MIM 600253) (Mayer et al., 2019). To date, only one gene has been associated with IIN, the *FRMD7* gene, located in the Xq26 chromosome. X-linked mutations in the *FRMD7* gene are the major known cause of familial IIN and idiopathic infantile periodic alternating nystagmus (Tarpey et al., 2006; Watkins et al., 2012). The *AHR* gene mutation in our patients results in a premature stop codon replacing a glutamine codon at positon 621 (out of 848 for the wild type protein) and thus, is expected to result in a truncated protein lacking a significant part of the C-terminus, including the Q-rich domain. This domain was recently shown to play a role in regulation of intracellular trafficking of the AHR, in the context of both nucleocytoplasmic shuttling and receptor activation. It was shown that the section between residues 648–661, which is in close proximity to the reported mutation, is necessary and sufficient to facilitate ligand-induced nuclear accumulation of the AHR and subsequent transcriptional activation (Kumar et al., 2001; Tkachenko et al., 2016). Recently, another family of Indian origin was described whose members carry a unique splicing mutation in the Q-rich domain of AHR. In this instance, the mutant AHR is truncated at approximately amino acid 400 which would make it even shorter than the mutant AHR in our family (Zhou et al., 2018). It is noteworthy that the homozygous mutant members in this family presented with a Retinitis pigmentosa-like retinal degeneration while any pupillary movements or morphological development of the fovea were not investigated in this study.

Investigation of Ahr null mice (Ahr −/−) revealed that these animals were afflicted by horizontal nystagmus similar to that observed in our patients (Chevallier et al., 2013). AHR knockout mice (AHR-KO) were shown to possess an impaired optic nerve myelin sheath and exhibited modifications in lipid composition as well as in the expression of myelin-associated glycoprotein (MAG) (Juricek et al., 2017; Shackleford et al., 2018). Supporting the involvement of lipids in this pathology, AHR was recently shown to activate the promoter for a small subunit of the serine palmitoyltransferase, the first and rate-limiting step in sphingolipid synthesis, in both mice and HeLa cells (Majumder et al., 2020).

Aryl hydrocarbon receptor is a highly conserved protein of the basic helix–loop–helix (bHLH)-PAS (PER-ARNT-SIM) family that acts as a ligand-activated transcription factor (Bock and Köhle, 2006; Barouki et al., 2012; Bock, 2013, 2014; Zhou, 2016; Nebert, 2017). This receptor was shown to play a role under normal physiological conditions in a multitude of processes, such as immunity, inflammation, neurogenesis, and tumorigenesis (Bock, 2017; Kawajiri and Fujii-Kuriyama, 2017). The non-ligand bound AHR exists in the cytosol as part of a multiprotein complex containing heat shock protein 90 (hsp90), a 38-kDa AIP (AHR-interacting protein), and a less well characterized protein, the co-chaperone p23 (Denison et al., 2011), which maintains AHR in a ligand-binding conformation and prevents its nuclear translocation and/or dimerization with ARNT (Kewley et al., 2004). Ligand binding leads to a conformational change in the AHR which allows nuclear translocation and dimerization with ARNT (AHR nuclear translocator). AHR-ARNT dimerization causes dissociation of the AHR from its accompanying chaperones (Tsuji et al., 2014) and converts the AHR into a high affinity DNA-binding form Soshilov and Denison (2011). The heterodimeric AHR-ARNT interacts with xenobiotic-response elements (XREs) and upregulates transcription of xenobiotic-metabolizing enzymes such as the cytochrome P450 genes (e.g., CYP1A1 and CYP1B1) as well as the phase II enzymes (Whitlock, 1999; Abel and Haarmann-Stemmann, 2010; Bock, 2014; Nebert, 2017).

The goal of our work is to investigate the biochemical consequences of the nystagmus-linked p.Q621^∗^ AHR mutation. Although it was hypothesized that the protein might be degraded due to non-sense mediated mRNA decay (Mayer et al., 2019), this was never examined. In the present study, as a step toward understanding the disease mechanism, we examined the expression of the *AHR* gene and protein in leukocyte culture lysates from patients. Our results show that the AHR mutant is stably expressed at both the mRNA and protein levels, despite lacking a C-terminus. We further examined the expression of *AHR* target and *AHR*-linked genes in leukocytes from healthy controls, heterozygous subjects and homozygous patients upon induction with the known AHR ligand benzo(a)anthracene. Overall, we found a reduction in the expression levels of mRNA and protein of known AHR targets that was consistent with the patient genotype. This work presents the first biochemical analysis of this affected family, a unique “human model” for AHR dysfunction, which can help to further investigation of the multiplicity of AHR-related pathways.

## Materials and Methods

Blood samples from three patients and two unaffected heterozygotic family members, as well as samples from five healthy volunteers, were collected at the Hillel Yaffe Medical Center, Hadera, Israel, in accordance with the tenets of the Helsinki Declaration. The study was carried out at Tel-Aviv University and was approved by the Tel-Aviv University ethics committee. Written informed consent was obtained from participants in this study or from their legal guardians.

### Leukocyte Isolation

Peripheral blood (15–20 ml) was collected from healthy and affected subjects. The blood was diluted twice in PBS and layered onto Ficoll-Paque Plus sterile solution. Centrifugation was carried out at 400 × *g* for 25 min at RT. The lymphocyte-rich fraction was collected for the subsequent steps.

### Primary Lymphocyte Cell Culture

Primary lymphocyte cell cultures were established as described in Gurtoo et al. (1975) and Atlas et al. (1976). The previously collected lymphocyte-rich fraction was diluted in RPMI 1640 growth medium with L-glutamine supplemented with heat-inactivated Fetal Bovine Serum (FBS), and penicillin/streptomycin. The lymphocyte pellet was resuspended in growth medium containing the mitogen Phytohemagglutinin-M (PHA) and cells were counted and plated in T25 cell culture flasks at a density of 2–4 × 10^6^ cells per ml. After incubation at 37°C, 5% CO_2_ for 48 h, benzo(a)anthracene (BA) in acetone was added to a final concentration of 5 μM for AHR induction and acetone was added to control flasks as a vehicle control. After 24 h of further incubation, the cultures in each treatment group were separately harvested. The cells were usually assayed immediately; otherwise, centrifuged cell pellets were stored at −80°C for up to 1 month in frozen growth medium containing 10% DMSO. The total RNA extraction was carried out from freshly collected cells. Protein concentration was determined by the Bicinchoninic Acid (BCA) assay with reagents purchased from Sigma and BSA was used as a calibration standard (Walker, 2009).

### Quantitative RT PCR

The work was carried out on cultivated human lymphocytes as described in Lin et al. (2003). We used quantitative real time polymerase chain reaction (qRT-PCR) to quantitate the relative mRNA expression of *AHR*, Cytochrome P450-1A1 (*CYP1A1*), P450-1B1 (*CYP1B1*), *TIPARP* (TCDD-inducible poly) (ADP-ribose) polymerase, *ARNT* (AHR Nuclear Translocator) and *AHRR* (AHR repressor). The quantitative RT-PCR included *GAPDH* (glyceraldehyde-3 phosphate dehydrogenase) as an internal standard. Relative expression of all mRNAs of interest was determined in naïve (unstimulated) as well as benzo(a)anthracene stimulated lymphocyte cultures. All measurements were performed in triplicate and standardized to the level of *GAPDH* expression. The expression level of target genes in uninduced lymphocyte cultures from healthy volunteers was used as a reference value for calculation of target gene expression in treated cultures. The relative expression level of the target gene, 2^–ΔΔ^Ct, was calculated using the method developed by Livak and Schmittgen (2001), with StepOne Software v.2.3 (Applied Biosystems Thermo Fisher Scientific).

Total RNA was prepared using the PureLink RNA Mini Kit (Invitrogen) according to the manufacturer’s protocol. The RNA quantity and quality was measured in a NanoDrop Spectrophotometer. 4–5 μg of total RNA was reverse transcribed to cDNA using Oligo(dT) and the SuperScript^TM^ First-Strand Synthesis System for RT PCR (Invitrogen) according to the manufacturer’s protocol. Quantitative PCR was performed using the qPCRBIO Fast qPCR SyGreen Blue Mix, Hi-ROX (PCRBIOSYSTEMS) on a StepOnePlus qRT PCR apparatus (Applied Biosystems).

Primers were designed using the NCBI Primer Blast program for finding highly specific primer pairs for each human gene sequence taken from the NCBI gene library. The specificity of each primer pair was tested by standard curve calibration and melting curve analysis to confirm reliable reaction efficiency and the appearance one single product for each gene of interest. The resultant products were analyzed by agarose gel electrophoresis to confirm the appropriate predicted size of the PCR product (data not shown). Primer pairs used in this work are listed in Table 1.

**TABLE 1:** Primer sequences for quantitative real time PCR.

Genes (Accession No.)	Sequences	Product Length
*GAPDH* (NM_001256799)	FW: 5′-AATATGATTCCACCCATGGC-3′	120
	REV: 5′-CCCACTTGATTTTGGAGGGA-3′	
*AHR*	FW: 5′-CAACAGCAACAGTCCTTGGC-3′	133
(NM_001621)	REV: 5′-GCTTCATCTTCTGACACAGC-3′	
*CYP1A1* (NM_000499)	FW: 5′-CTACCTACCCAACCCTTCCCT-3′	243
	REV: 5′-ACTGTGTCAAACCCAGCTCCAA-3′	
*CYP1B1* (NM_000104)	FW: 5′-GGCAGAATTGGATCAGGTCGT-3′	247
	REV: 5′-GTTCTCCGGGTTAGGCCAC-3′	
*TIPARP* (NM_015508)	FW: 5′-GGAGAGAGTATCCCGAGTCTGT-3′	195
	REV: 5′-GAACCCCACCAAGTGTCTGT-3′	
*AHRR* (NM_020731)	FW: 5′-TGATGCTATCCTGGGGAGGC-3′	188
	REV: 5′-TCATGAGTGGCTCGGGACAG-3′	
*ARNT* (NM_178427)	FW: 5′-CCCTCCCAGATGATGACCCA-3′	214
	REV: 5′-AAGAGTTCCTGTGGCTGGTAG-3′	

### Protein Expression Level of AHR and AHR-Related Proteins

The protein expression levels of AHR, CYP1A1, CYP1B1, TIPARP, ARNT, and AHR-R were estimated by Western Blot analysis. Protein extraction from non-induced and BA-induced leukocyte cultures was achieved by cell lysis in buffer containing: 2% Triton-X-100; 1% SDS; 100 mM NaCl; 10 mM Tris pH 8.0; 1 mM EDTA and 1 mM EGTA supplemented by phosphatase inhibitors 2 mM sodium orthovanadate and 1 mM K-Na tartrate, protease inhibitor cocktail (Roche), 0.2 mM PMSF, 4 mM MgCl_2_ and DNase. After a 30 min incubation of the resuspended leukocyte pellets with lysis buffer on ice, the supernatants were separated from insoluble aggregates by centrifugation. The protein concentration of the supernatant was measured using the Sigma BCA kit with BSA as a standard calibration curve. Total protein (30–50 μg per lane) was loaded on 10% SDS PAGE and proteins were separated at 100 V for 20 min and then at 130 V for 60–65 min. The proteins were transferred to a nitrocellulose membrane by the *Trans*-Blot Turbo transfer system (Bio-Rad). The proteins of interest were detected with specific primary antibodies: Anti-Aryl hydrocarbon Receptor antibody (anti-AHR) AhR (D5S6H) Rabbit mAb C-terminal, (CST 83200), Anti-Aryl hydrocarbon Receptor antibody [RPT1] ChIP Grade N-terminal (ab2770, Abcam), Anti-Cytochrome P450 1A1 antibody, (ab3568, Abcam), Anti-CYP1B1 antibody [EPR14972]–C-terminal (ab185954, Abcam), anti-ARNT–HIF-1β/ARNT (D28F3) Rabbit mAb (CST, 5537), anti-TIPARP, PARP7 antibody (ab200390, Abcam), Anti-AHRR antibody (ab108518, Abcam), as a loading control Mouse Anti-β-Actin (C4), (sc-47778, Santa Cruz, CA, United States) or Rabbit Anti-MAP Kinase (MAPK, ERK-1/2) (Sigma M 5670) were used. Infra-Red 700 or 800 nm secondary anti-rabbit or anti-mouse were used for protein determination. The specific bands were visualized by scanning on an Odyssey Infrared Li-Core Bioscience scanner. The quantitation was done with Odyssey comprehensive software V.3.

### Enzymatic AHH Activity Measurements

The aryl hydrocarbon hydroxylase (AHH) activity was measured by the fluorometric method described for human cultured lymphocytes by Gurtoo et al. (1975) with modifications made in Atlas et al. (1976). Briefly, the incubation mixture consisted of 50 mM Tris–HCl, pH 8.5, 0.36 mM NADPH, 0.42 mM NADH (4.2 mM), 3 mM MgCl_2_, 0.7 mg/ml BSA, 0.2 M sucrose, and DDW, and 10–15 μl lymphocytes (∼2.5 mg/ml, ∼5 × 104 cells/ml) in a total volume of 0.25 ml. The reaction was initiated by adding 2 mM benzo(a)pyrene followed by incubation at 37°C in a shaking incubator for 1 h. The reaction was stopped by the addition of 0.75 mL cold acetone:hexane (1:3) mixture. Aliquots of 0.25 ml were taken from the organic upper phase [containing the Hydroxy-Benzo-Pyrene (OH-BP) reaction product], added to 0.75 ml 1 M NaOH. The lower fraction containing water soluble OH-BP was transferred in a new Eppendorf tube. Fluorescence emission was measured at 596 nm under excitation at 515 nm using a Horiba Jobin Yvon FL3-11 Spectrofluorometer and supplied software. The AHH activity was expressed in picomol OH-BP/min/mg protein.

### Statistical Analysis

Results are presented as means of at least three independent experiments. Data are expressed as means ± SD. Statistical significance of the differences was assessed using paired Student’s *t*-test. *p* < 0.01 or *p* < 0.05 were the criteria of significance.

## Results

### mRNA and Protein Expression of AHR in Patients

In the mutant AHR gene of our patients, the stop codon is located at amino acid 621, which should yield a truncated protein lacking a significant part of the C-terminus. We speculated that the mutant AHR may fail to be stably expressed, like many truncated proteins, as part of the cell’s sophisticated mRNA quality control apparatus (Karamyshev and Karamysheva, 2018). Indeed, the phenotype of our patients is similar to that of mice harboring a complete AHR knockout, suggesting that the mutant AHR of our patients might indeed be unstable and degraded at either the mRNA or/and protein level. Table 2 and Supplementary Figure S1 show that the relative quantity of *AHR* mRNA transcripts in heterozygotes (Het) is about 70% that of healthy subjects (WT) whereas *AHR* mRNA expression in homozygous patients (Hom) is about 40% of the level in WT. These differences most likely result from partial mRNA decay of mutant transcripts. Thus, despite the stop codon mutation, the mutant *AHR* mRNA is stably expressed in the leukocytes of patients albeit at reduced levels. As would be expected, similar values were obtained when the samples were taken from leukocyte cultures that were treated with BA, as BA is not expected to induce the mRNA of *AHR* itself rather, expression of its target genes.

**TABLE 2:** Relative mRNA expression of *AHR* and *AHR*-target genes.

Gene	WT	WT + BA	Het	Het + BA	Hom	Hom + BA
*AHR*	1 ± 0.089	0.84 ± 0.15	0.73 ± 0.13	0.62 ± 0.19	0.37 ± 0.12	0.34 ± 0.14
*CYP1A1*	1 ± 0.16	8.16 ± 1.96*	0.71 ± 0.22	0.87 ± 0.22	0.26 ± 0.08	0.22 ± 0.07
*CYP1B1*	1 ± 0.08	2.26 ± 0.16*	1.05 ± 0.14	1.61 ± 0.15*	0.32 ± 0.17	0.19 ± 0.13
*TiPARP*	1 ± 0.12	2.61 ± 0.25**	1.4 ± 0.35	1.21 ± 0.22	0.79 ± 0.12	0.62 ± 0.15
*ARNT*	1 ± 0.15	0.94 ± 0.17	1.21 ± 0.29	1.26 ± 0.10	1.81 ± 0.27	1.60 ± 0.26
*AHRR*	1 ± 0.29	0.81 ± 0.21	0.96 ± 0.31	0.91 ± 0.36	1.26 ± 0.25	1.10 ± 0.31

The data are represented as Relative Quantity (RQ) calculated by the RT PCR instrument software. The left column lists the tested genes. Patient genotype is described in column headings: Het – heterozygous subjects n = 2); Hom – homozygous patients (n = 3). Gene expression levels are normalized to the level of healthy control (WT) with GAPDH as an internal control. The data represent averages of three technical replicates for each of three healthy controls, two heterozygous subjects (Het), and three homozygous patients (Hom) for untreated and BA treated (+BA) leukocyte cultures. The data are shown as the mean of three replicates for each patient ±SD. A two-tailed t-test analysis of statistical significance was done; p < 0.01* and p < 0.05** refer to BA induced vs. untreated samples.

**FIGURE 1:**
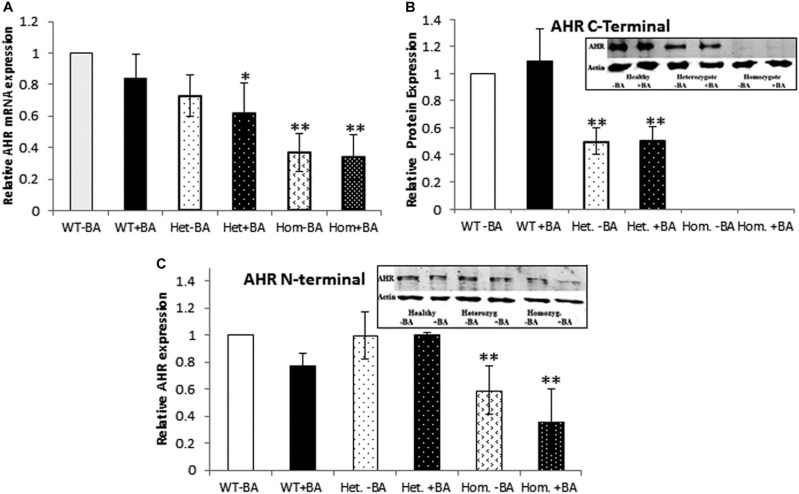
*AHR* mRNA **(A)** and protein **(B,C)** expression. In all graphs, the columns correspond to lysates of non-induced leukocyte cultures (white columns) or lysates of BA-induced cultures (BA, black columns). **(A)** Relative *AHR* mRNA expression was examined by qRT PCR using *GAPDH* as an internal control. **(B)** AHR protein expression was detected by western blotting and detection with an antibody against a C-terminal epitope. **(C)** AHR protein expression was detected by western blotting and detection with an antibody against an N-terminal epitope. In all experiments, leukocyte culture lysates were run on 10% SDS PAGE, transferred to nitrocellulose and probed with specific AHR C-terminal **(B)** or N-terminal **(C)** antibodies. Band densities were normalized to β-Actin and quantified as a fraction of non-treated WT samples. Data were obtained separately from either patients or healthy subjects and were averaged. Data are shown as mean ± SD, *n* = 4 technical replicates for each of three healthy volunteers (Heal), two heterozygous patients (Het), and three homozygous patients (Hom). Insets show a representative AHR western blot obtained using a. LI-COR Odyssey IR scanner. Upper bands correspond to AHR and lower bands correspond to the β-actin loading control. A two-tailed *t*-test analysis of statistical significance was done; *p* < 0.01* and *p* < 0.05** refer to variances between homozygous patients vs. healthy control samples.

The fact that we detected *AHR* mRNA expression (Figure 1A) in homozygous patients, does not necessarily imply that the protein is stable expressed. Consequently, we used western blot analysis to estimate protein expression levels in lysates prepared from BA treated and untreated leukocyte cultures. We examined whether the AHR protein is expressed in lysates of cells taken from of healthy controls, heterozygotes and homozygous patients.

**FIGURE 2:**
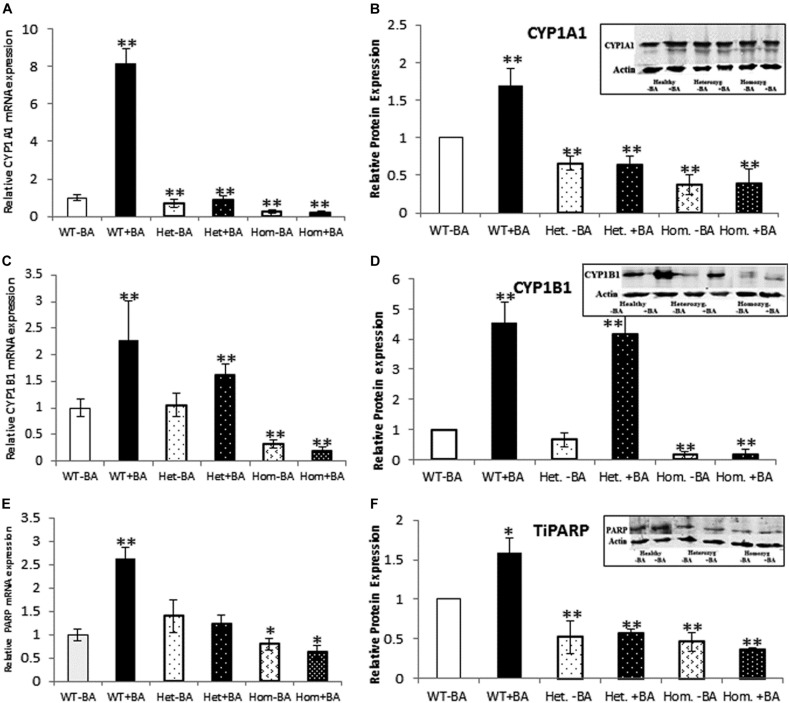
mRNA and protein expression of *AHR* target genes. CYP1A1 **(A,B)**, CYP1B1 **(C,D)**, and TiPARP **(E,F)**. In all graphs, the columns correspond to lysates of non-induced leukocyte cultures (white columns) or lysates of BA-induced cultures (BA, black columns). **(A)** Relative *CYP1A1* mRNA expression was examined by qRT PCR using *GAPDH* as an internal control. **(B)** Relative CYP1A1 protein expression was determined by western blotting with an antibody against CYP1A1. **(C)** Relative *CYP1B1* mRNA expression was examined by qRT PCR with *GAPDH* as internal control. **(D)** CYP1B1 protein expression was determined by western blotting with CYP1B1 antibodies. **(E)** Relative *TiPARP* mRNA expression was examined by qRT PCR using *GAPDH* as internal control. **(F)** TiPARP protein expression was detected with PARP antibodies. The leukocyte culture lysates were run on 10% SDS PAGE, transferred to nitrocellulose and probed with specific antibodies as described in the “Materials and Methods” section. Band densities were normalized to the β-Actin loading control and quantified as a fraction of non-treated WT. Data were obtained separately from either patients or healthy subjects and were averaged. Data are shown as mean ± SD, *n* = 4 technical replicates for each of three healthy volunteers, two heterozygous patients (Het), and three homozygous patients (Hom). Insets show a representative western blot obtained using a LI-COR Odyssey IR scanner. Upper bands correspond to tested protein and lower bands correspond to β-Actin loading control. A two-tailed *t*-test analysis of statistical significance was done; *p* < 0.01* and *p* < 0.05** refer variances between homozygous patients vs. healthy control samples.

To this end, we used two different antibodies for AHR immunodetection (Figures 1B,C). First, we carried out immunodetection with antibodies raised against the C-terminal domain of AHR. Since the mutant AHR has a stop codon at amino acid 621, it should be lacking its C-terminus. Consistent with this prediction, samples taken from homozygous mutant patients lacked any discernible band of AHR expression whereas heterozygotes expressed about half the amount of healthy controls (Figure 1B). In order to examine whether the truncated protein is stable or undergoes rapid degradation, we used an antibody against an N-terminal epitope of AHR that should be able to detect both the full-length and the truncated forms of AHR. As seen in the Figure 2C AHR bands are detected in all tested samples, indicating that the truncated AHR protein exhibits significant stability in the lymphocytes. The level of AHR in heterozygous subjects was similar to that of healthy subjects, whereas homozygous patients had about half the amount found in healthy and heterozygous subjects. These results are in agreement with the mRNA expression pattern presented herein and AHR protein expression detected with C-terminus antibody. Interestingly, the mobility of the truncated protein of homozygous patients is similar to that of wild type AHR. It should be noted that anomalous migration of proteins in PAGE is a known phenomenon, explained by the ability of various physical characteristics such as acidity (Rath et al., 2009) or hydrophobicity (Tiwari et al., 2019), or helix-loop-helix (“hairpin”) sequences (Shirai et al., 2008) to influence the binding of SDS.

### mRNA and Protein Expression Levels of AHR Target Genes

As a starting point for investigating the effects of the mutation, we carried out a quantitative expression analysis for some of the main AHR target genes *CYP1A1*, *CYP1B1*, and *TiPARP* [TCDD Inducible Poly(ADP-Ribose) Polymerase (Qiang et al., 2001), a less known AHR target that may contribute to AHR feedback regulation]. The tests were carried out on the same lysates extracted from lymphocyte cultures that were taken from patients and healthy controls, as described in the first section. We found that basal mRNA and protein expression levels of CYP1A1 and CYP1B1 in homozygous mutant patients were significantly lower than in healthy volunteers, reaching ∼26 and 32% of the healthy control average, respectively (Figures 2B,D). Basal mRNA expression levels of these targets in heterozygotes were similar to levels in healthy controls (see Table 2 and Figures 2A,C). In addition, we examined the induction of AHR target genes in lymphocytes that had undergone induction by benzo[a]anthracene (BA) treatment (Table 2 and Supplementary Figure S1). Lymphocytes from healthy controls showed eight-fold and ∼2.5-fold increases in *CYP1A1* and *CYP1B1* transcript levels, respectively, whereas the homozygous mutant patients exhibited no increase but rather a slight decrease in *CYP1A1* and *CYP1B1* transcript levels suggesting a failure of AHR to induce its downstream targets. A lack of activation in homozygous patients is expected, considering the location of the mutation, which is found in the transcription activation region (Tkachenko et al., 2016). This result is also consistent with the lack of activation observed in AHR deficient mice (Gonzalez et al., 1995; Fernandez-Salguero et al., 1996; Mimura et al., 1997; Shimizu et al., 2000; Bunger et al., 2008). Surprisingly, the heterozygous patients also showed no induction of *CYP1A1* expression in response to AHR activation, despite carrying one wild-type allele. Similarly, no induction was observed for AHR-induced *CYP1B1* gene and protein expression for homozygous patients. However, heterozygous patients demonstrate clear inducibility of *CYP1B1* mRNA expression, a 1.6-fold increase over uninduced samples. This compares to a 2.3-fold increase for lysates taken from treated wild type cultures (from healthy controls) compared to uninduced wild type samples. In terms of CYP1B1 protein expression, a very strong increase (greater than four-fold) was observed for both healthy subjects and heterozygotes.

The expression of *TIPARP*, a third *AHR* target gene was also examined. We found that expression of this gene in homozygous patients was lower than in healthy controls (70% of the control) whereas heterozygous subjects demonstrated 40% higher basal level of this gene vs. healthy controls, although this increase was within the error range (see Table 1 and Figures 2E,F). In contrast to healthy controls, neither hetero- nor homozygous patients exhibited BA-induction at either the mRNA or protein expression level. The fact that *CYP1A1* and *TiPARP* genes are not induced upon BA-activation of AHR in the heterozygotes, who do not exhibit the nystagmus phenotype, might suggest that their gene products are not involved in the ocular pathology observed in the homozygous patients. Thus, *CYP1B1* is the one gene among the three tested genes that behaves differently in heterozygotes, who don’t have the IIN phenotype, and homozygotes, who suffer from the eye disease.

### mRNA and Protein Expression Levels of AHR-Regulated Genes ARNT and AHR-R

**FIGURE 3:**
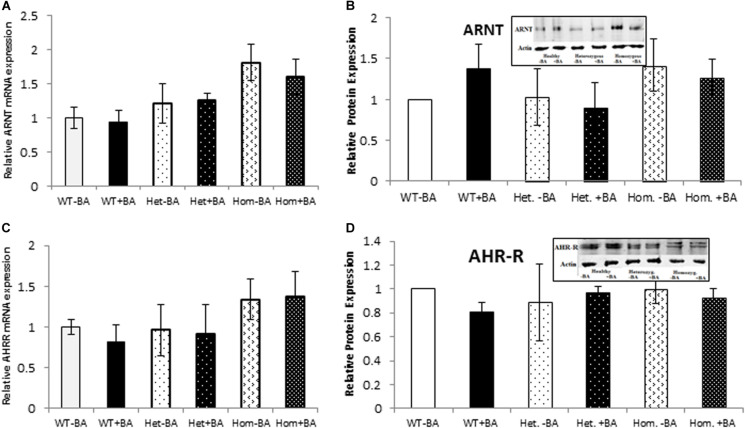
mRNA and Protein expression of *AHR* target genes. ARNT **(A,B)** and AHR-R **(C,D)**. In all graphs, the columns correspond to lysates of non-induced leukocyte cultures (white columns) or lysates of BA-induced cultures (BA, black columns). **(A)** Relative ARNT mRNA expression was examined by qRT PCR using *GAPDH* as an internal control. **(B)** ARNT protein expression was detected by western blotting and detection with ARNT antibodies. **(C)** Relative AHR-R mRNA expression was examined by qRT PCR using GAPDH as an internal control. **(D)** AHR-R protein expression was detected by western blotting and detection with AHR-R antibodies. The leukocyte culture lysates were run on 10% SDS PAGE, transferred to nitrocellulose and probed with specific antibodies as described in the “Materials and Methods” section. Bands were normalized to the β-Actin loading control and quantified as a fraction of non-treated WT. Data were obtained separately for either patient or healthy subject and were averaged. Data are shown as mean ± SD, *n* = 4 technical replicates for each of three healthy volunteers, two heterozygous subjects (Het), and three homozygous patients (Hom). Insets show a representative western blot obtained using a LI-COR Odyssey IR scanner. Upper bands correspond to tested protein and lower bands correspond to β-Actin loading control. A two-tailed *t*-test analysis of statistical significance was done; *p* < 0.01* and *p* < 0.05** refer variances between homozygous patients vs. healthy control samples.

We also examined the expression of two AHR-interacting genes, ARNT and AHR-R that, respectively activate or repress AHR itself. Mutation of AHR is not expected to affect induction of these genes. Indeed, for all tested groups, the BA treated samples show the same expression levels as the untreated samples for both proteins. Figure 3 shows mRNA (Figures A,C) and protein (Figures B,D) expression levels of ARNT and AHR-R. Neither of these mRNAs or proteins undergo activation when AHR is stimulated with BA. Basal levels of ARNT and AHR-R in heterozygous patients are similar to the levels found in healthy subjects. In contrast, the levels of ARNT and AHR-R in homozygous patients are slightly but significantly higher compared with healthy and heterozygous subjects, perhaps representing some kind of compensation mechanism for impaired levels or function of AHR. These differences were observed for mRNA expression, but not for AHR-R protein expression.

### Activity of Aryl Hydrocarbon Hydroxylase (AHH)

As an additional measure of AHR activation, we measured the AHH activity carried out by a number of the CYP P450 monooxygenase enzymes, including CYP1A1, 1A2, and 1B1 (Liu et al., 2013), in the same leukocyte lysates as described above. Activity was determined immediately after lymphocyte culture harvesting and repeated during the course of 1 month using the frozen lymphocytes. The average AHH activity (3–6 technical replicates for each lymphocyte sample) was calculated (see Table 3, WT). The basal AHH activity of patients was lower than that of healthy controls, with the heterozygous patient exhibiting approximately 30% activity of healthy controls and homozygous patients exhibiting less than 20% of the wild type non-induced activity. Healthy controls displayed a 3.6-fold increase in AHH activity over the basal level, compared with only a 2.3-fold increase for the heterozygous patients and no significant increase over basal activity for homozygous patients upon induction with BA. These data are consistent with the patient genotype, with the heterozygous patient having intermediate basal and BA-induced AHH activity and homozygotic patients exhibiting minimal basal and BA-induced activity. The intermediate enzymatic activity induced by BA for heterozygous patients is in apparent contradiction with the mRNA and protein expression data. However, since the fluorometric method described in Gurtoo et al. (1975) and Atlas et al. (1976), measures the common hydroxylase activity, the contribution of each enzyme CYP1A1, CYP1A2, or CYP1B1 is not known.

**TABLE 3:** AHH activity in lymphocytes of patients vs. healthy controls.

Samples	AHH activity in pmol/min/mg protein	Fold activation after BA induction
WT^–^	3.37 ± 1.59	
WT^+^	12.13 ± 3.77*	3.6
Het^–^	0.92 ± 0.25	
Het^+^	2.09 ± 0.57**	2.3
Hom^–^	0.48 ± 0.20	
Hom^+^	0.58 ± 0.22	1.2

A two-tailed t-test analysis of statistical significance was done; p < 0.01* and p < 0.05** refer to BA induced vs. untreated samples.

## Discussion

Aryl hydrocarbon receptor is a transcription factor that is known for its canonical role in the induction of detoxification enzymes upon exposure to xenobiotics and drugs. In addition, AHR was reported to be involved in a multitude of normal physiological function as well as pathological processes such as cell circle regulation, immune response, apoptosis, oxidative stress, cancer, tumorigenesis and CNS metabolism (Nebert et al., 2000; Marlowe and Puga, 2005; Puga et al., 2009; Juricek and Coumoul, 2018). Recently, two mutations in the AHR gene were shown to be associated with visual defects (Zhou et al., 2018; Mayer et al., 2019). The main goal of this study was to examine potential changes in mRNA and protein expression levels of canonical AHR targets in subjects carrying the stop mutation c.1861C > T; p.Q621^∗^ which is associated with foveal hypoplasia and infantile nystagmus (Mayer et al., 2019). For this purpose, we prepared lymphocytes from healthy heterozygous carriers and affected homozygous mutant patients and examined the expression of AHR, its partner and target genes, in naïve and benzo(a)anthracene (BA) induced cells. Using this readily available system, we were able to successfully examine the expression of *AHR* and some of its known targets and partners, although this system might have certain limitations in terms of investigating tissue-specific and developmental gene expression.

Since it was hypothesized that the AHR mRNA might be degraded due to non-sense mediated decay, we examined first whether the truncated form of AHR is expressed in lymphocytes prepared from the peripheral blood of patients vs. healthy controls. Our results, both at the level of mRNA and protein expression, exclude the possibility of strong downregulation or degradation of AHR, as the AHR was detected in both homo- and heterozygous cells harboring the stop mutation. Further experiments showed that the full-length AHR protein was expressed approximately 50% in heterozygous patients compare to healthy volunteers, corresponding to the presence of one mutant and one wild type allele in the heterozygous genotype. Using AHR antibodies raised against the N-terminus, we observed AHR bands in all subjects at approximately the same mobility. This fact may be explained by abnormal electrophoretic SDS mobility which can be affected by a number of physical characteristics such as acidity, hydrophobicity and helical hairpin structures, as was most recently discussed (Shirai et al., 2008; Rath et al., 2009; Tiwari et al., 2019). Indeed, it was shown that approximately 40% of the yeast proteome does not migrate as would be expected based on the calculated molecular weight of the proteins (Tiwari et al., 2019) and such discrepancies in calculated molecular weight can reach even 30% (Shirai et al., 2008; Tiwari et al., 2019). It is also possible that the truncated protein undergoes some post-translational modification such phosphorylation, glycosylation, or ubiquitination, etc. which can lead to anomalous electrophoretic mobility (Guan et al., 2015).

We also examined the effect of the mutation on the expression ARNT and AHR-R, two proteins that belong to the bHLH/PAS family and that regulate AHR transfer from the cytoplasm to the nucleus (Mimura et al., 1999; Kikuchi et al., 2003; Evans et al., 2008). No change in protein expression was observed in any of the samples from patients or healthy volunteers. In fact, the mRNA level of these genes was increased in homozygous cells, possibly as compensation for loss of ARH transcription factor activity.

Expression of three AHR target genes, *CYP1A1*, *CYP1B1*, and *TiPARP* was also investigated. As expected, none of the three genes was induced upon BA treatment of lymphocytes prepared from homozygous mutant patients having two mutant alleles. The heterozygous subjects have one mutant allele and exhibit no nystagmus, as expected for a recessive gene. Interestingly, despite the presence of one wild type allele, there is no BA activation of the *CYP1A1* and *TiPARP* targets in heterozygotes. Thus, the mutant AHR function seems to behave in a dominant manner, suppressing the function of the wild type allele, without affecting phenotype. One possible explanation for this phenomenon could lie in the different ability of the mutant AHR to dimerize with itself and other factors as well as to interact with DNA-binding sites. For example, it could form irreversible, non-functional dimers with ARNT, effectively sequestering this transcription partner from the wild type AHR. Moreover, it was shown that regulatory regions in the DNA can influence the types of dimers formed for some helix–loop–helix (bHLH) proteins (Inamoto et al., 2017).

The fact that two proteins, CYP1A1 and TiPARP, are not induced in the heterozygous patients who lack the Nystagmus phenotype, indicate that these genes are not involved disease development. Notably, the only target that was found to be differentially expressed in heterozygous compared to affected homozygous patients, CYP1B1, was reported to be associated with congenital glaucoma (Vasiliou and Gonzalez, 2008). Our patients did not exhibit any sign of glaucoma and no signs of glaucoma were reported in AHR-KO mice. Future investigation is required to elucidate whether CYP1B1 protein is involved in development of disease manifestation in the AHR patients.

Finally, measured AHH activity was shown to be considerably lower in both hetero- and homozygous patients compared to healthy volunteers (Table 3). Upon BA activation AHR no induction was observed in homozygous patients. The heterozygous patients show intermediate induction of the AHH activity, but its inducibility was significantly lower than revealed healthy subjects. This is inconsistent with the total lack of induction seen at the mRNA and protein levels and might be explained by the fact that the method used to measure enzyme activity detects the combined activity of several cytochrome P450 monooxygenases.

It was recently published that AHR-KO are afflicted by a nystagmus and found to have an impaired optic nerve myelin sheath along with modifications in lipid composition and in the expression of MAG (Juricek et al., 2017; Shackleford et al., 2018). Experiments to determine MAG levels in lymphocyte cultures obtained from the blood of the patients yielded very low cDNA copy numbers, so we were not able to confirm this finding in our patients. Recently, AHR was found to bind and activated the gene promoter of serine palmitoyltransferase small subunit A (SPTSSA), which encodes a subunit of the serine palmitoyltransferase that catalyzes the first and rate-limiting step in *de novo* sphingolipid biosynthesis (Majumder et al., 2020). Thus analysis of genes related to lipid metabolism would constitute a logical next step in our studies.

Overall, our results show that CYP1B1, at both the gene and protein level, undergoes induction in heterozygotes but not in homozygotes, making it a candidate to be involved in disease development. A recent study showed that activation of AHR induces changes in the expression patterns of 158 different genes (Liamin et al., 2018). We are interested in identifying additional genes that are induced in heterozygotes, but not in homozygous patients, and believe that such an analysis can give us clues in identifying pathways involved in the development of the observed ocular pathologies.

## Data Availability Statement

The raw data supporting the conclusions of this article will be made available by the authors, without undue reservation.

## Ethics Statement

The studies involving human participants were reviewed and approved by Tel-Aviv University Ethics Committee, Permission #14221241_20190326. Written informed consent to participate in this study was provided by the participants’ legal guardian/next of kin.

## Author Contributions

AA, BW, CW, and NB conceived and designed the project. MM and RS provided the clinical samples and analysis. NB and CW performed experiments and wrote the manuscript. MR assisted in design of mRNA expression experiments. AA and BW interpreted the data and revised the manuscript. All authors read, revised and approved the final manuscript.

## Conflict of Interest

The authors declare that the research was conducted in the absence of any commercial or financial relationships that could be construed as a potential conflict of interest.
