# Supplementary figures and images for: Gene and Protein Expression in Subjects With a Nystagmus-Associated AHR Mutation
Source: Front Genet. 2020 Sep 24;11:582796. doi: 10.3389/fgene.2020.582796 (PMC7542227; doi:10.3389/fgene.2020.582796)

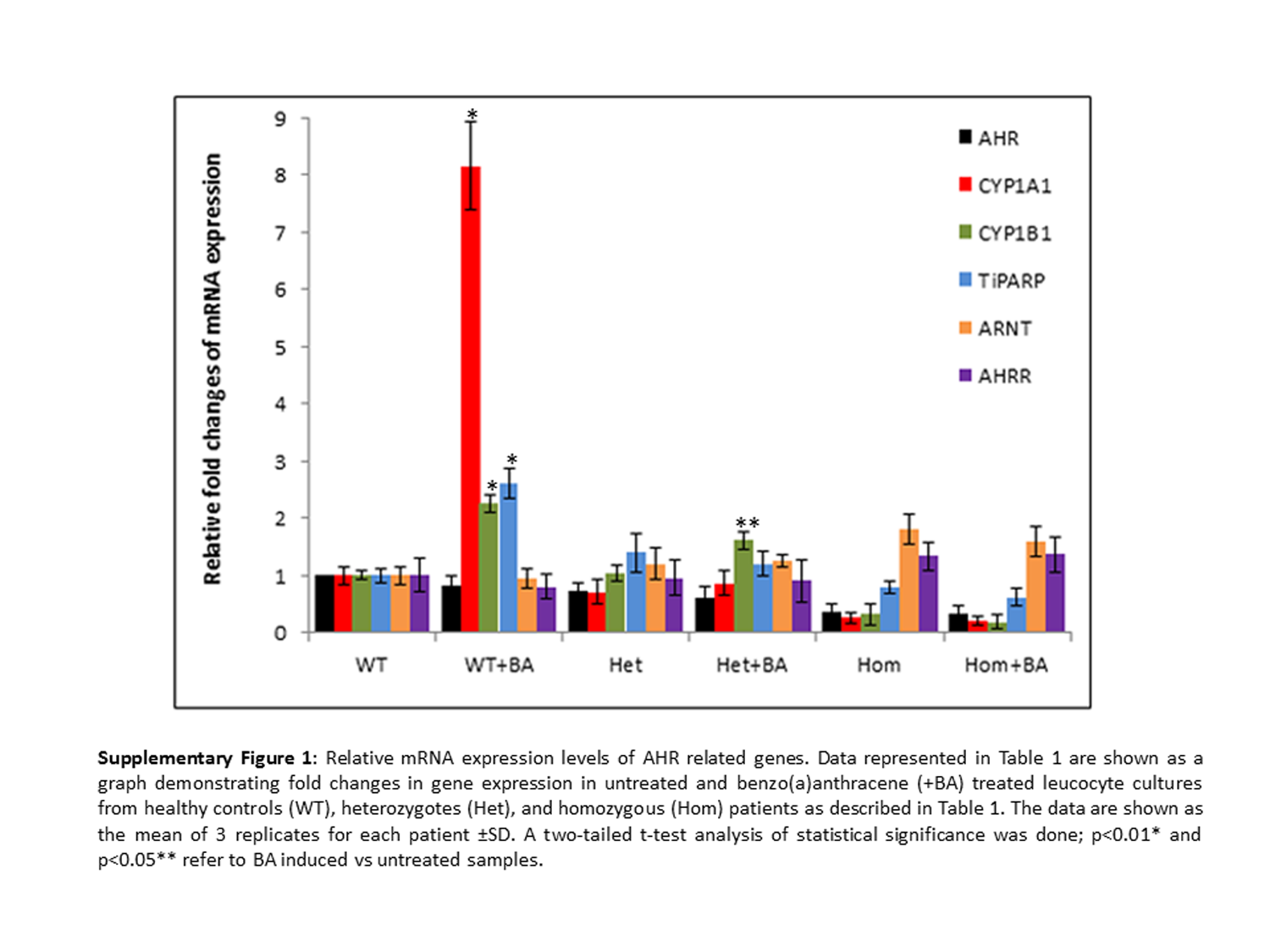

Supplement: Supplementary file 1 [file Image_1.tif]
